# Parents with mental health problems and their children in a German population based sample: Results of the BELLA study

**DOI:** 10.1371/journal.pone.0180410

**Published:** 2017-07-03

**Authors:** Angela Plass-Christl, Anne-Catherine Haller, Christiane Otto, Claus Barkmann, Silke Wiegand-Grefe, Heike Hölling, Michael Schulte-Markwort, Ulrike Ravens-Sieberer, Fionna Klasen

**Affiliations:** 1 Department of Child and Adolescent Psychiatry, Psychotherapy, and Psychosomatics, University Medical Center Hamburg-Eppendorf, Hamburg, Germany; 2 Department of Epidemiology and Health Monitoring, Robert Koch-Institute, Berlin, Germany; Chiba Daigaku, JAPAN

## Abstract

**Background:**

Mental health problems (MHP) of parents are associated with an increased risk of psychological and developmental difficulties in their children. This study aims at analyzing population-based data of parents with MHP and their children and the effects of associated risk factors in order to further targeted preventive and therapeutic interventions.

**Methods:**

The BELLA study is the mental health module of the German National Health Interview and Examination Survey among Children and Adolescents. MHP in parents and in their children as well as associated risk factors were examined in a sample of *N* = 1158 parents with children aged 11 to 17 years.

**Results:**

Parental MHP were identified in 18.6% of the sample. Risk factors associated with parental MHP were low SES, parental unemployment, stressful life events, parental daily strain, parental chronic disease, and child MHP. A rate of 19.1% of the children of parents with MHP reported MHP themselves, the corresponding rate among children of parents without MHP was 7.7%. In multiple regression analyses the risk for children of parents with MHP to report MHP themselves was almost two times higher than the risk of children of parents without MHP. Other significant associations with child MHP included gender, the parents’ age, and stressful life events.

**Conclusions:**

Parental MHP constitute a significant risk for the mental health of their children. Targeted screening methods and preventive interventions are needed.

## Introduction

Children of parents with mental health problems (MHP) have an increased risk of psychological and developmental difficulties compared to children of parents without MHP [1]. In clinical child and adolescent samples, previous studies have estimated that up to half of the children who undergo treatment for psychiatric illnesses have parents who have been diagnosed with a mental disorder [2,3]. Correspondingly, results of a meta-analysis suggest that more than half of the children of parents with mental disorders will develop MHP during their childhood or adolescence [4]. Moreover, MHP of parents show a substantial association with MHP of their children in late adulthood [5]. To date, population-based data regarding the situation of the risk group of children of parents with MHP have only rarely been examined [6], impeding the devise of appropriate public health policies.

For the etiology of mental health disorders biological and genetic factors are relevant [7]. In addition, the presence of psychosocial risk factors plays an important role in the development of MHP [8]. This is of particular relevance for children of parents with a mental illness [3,9]: For one, risk factors tend to cluster together [8–10]. Second, this applies even more to families with parental MHP, where almost all sources of psychosocial stress raising the probability for MHP seem to be present.

The few existing empirical findings based on population-based data regarding children of parents with MHP demonstrate that parental MHP are a significant predictor for poor child mental health as shown in a previous work based on data of the BELLA study in children aged 11 to 17 years [3,10]. Similarly, McLaughlin and colleagues [11] demonstrated that parental psychiatric disorders are significant predictors of the lifetime onset of psychiatric disorders in their children. Moreover, the general risk for children to develop any psychiatric disorder was shown to be 1.8 to 2.9 times higher compared with the total population if one parent was affected, rising to a risk of 2.2 to 4.6 times higher if both parents are affected [11].

Risk factors for the development of MHP in general include sociodemographic factors such as female gender, low socioeconomic status, [12,13], or unemployment [14]. Other risk factors for MHP are stressful live events (e.g., death of a close person or separation from a partner) [15], high strain in day-to-day life (e.g., tending a family member in need of care, problems related to work-life balance) [16], difficult family constellations (e.g., single parent, overcrowding of living quarters) [13,17], and physical health problems or chronic diseases [18]. For parents in particular, research has shown that the presence of MHP in their children add to the parent’s perceived burden [2,19].

The body of literature focusing on risk factors for child MHP is very similar. Along with child female gender, the family’s low socioeconomic status, parental unemployment [6,20], stressful live events, [10,21], parental strain in day-to-day life [10], difficult family constellations [6,22] have been associated with child MHP. Furthermore, parental chronic health problems [10,23] have also been shown to be significant risk factors for the development of child MHP.

The 12-months prevalence in Germany for adult mental disorders has been estimated to be 27.8% [12]. Data from the most current German micro census [24] estimates that 8.1 million families with approximately 13 million minor children lived in Germany in 2014. That is, potentially about 3.6 million children may live with a parent with a mental disorder. However, specific data concerning this risk group is lacking to date. Studies have shown that between a third and more than half of child mental health problems persist into adulthood [25,26]. Moreover, MHP heavily impact the individual’s life on the short- and long-term [27], as well as the family and the social environment [20]. Furthermore, MHP yield longstanding costs to society [27,28] and are responsible for an important percentage of the overall burden of disease [29].

Consequently, there is an urgent need for the design and implementation of targeted prevention and treatment strategies specifically designed for the risk group of children of parents with MHP; especially since studies have shown that the risk for MHP of children of parents with mental illnesses can effectively be reduced by 40% through preventive interventions [4]. Early intervention programs for children and adolescents moreover showed very good cost-benefit ratios and were qualified to be worth financing [30]. Thus, not only do preventive interventions for families with parents with MHP effectively reduce the risk for psychiatric morbidity in their children, they also show a good cost-effectiveness.

This study therefore set out with the main aim to examine the group of children of parents with MHP in a population-based sample in order to gain a better overview and deeper understanding of this particular risk group in Germany and the psychosocial risk factors associated with it. More precisely, we will 1) report how many children in Germany have at least one parent with MHP, 2) report the prevalence of children of parents with MHP that suffer from MHP themselves, 3) examine the effect of risk factors for mental health in parents, and 4) examine the effect of parental MHP on child mental health when controlling for other risk factors. Based on the body of literature, we expect risk factors for mental health (e.g., unemployment, chronic disease) to negatively affect parental mental health. We furthermore expect parental MHP to be one of the most important risk factors for child MHP.

## Methods

### Study design

Analyses for the present study were conducted using data from the 3^rd^ wave (2009 to 2012) of the Behaviour and Wellbeing of Children and Adolescents in Germany – the BELLA study. The BELLA study is the mental health module of the National Health Interview and Examination Survey for Children and Adolescents (KIGGS) in Germany and has been conducted in regular time intervals since 2003. KiGGS is a German representative survey that collects data on the health status of German children and adolescents from 0 to 17 years of age and into adulthood. A detailed description of the KiGGS sample, design, and methods has been published by Kamtsiuris, Lange, and Schaffrath [31]. The BELLA study wave 3 took part from 2009 until 2012 and collected data on mental health, risk and protective factors, health care, and sociodemographic data, using standardized and internationally tested instruments, if available. For further details regarding the design and methods of the BELLA study see [32] and [26].

### Measures

Parental MHP were measured using the German translation of the Symptom-Checklist Short version (SCL-S-9), a short form of the Symptom-Checklist-90-R [33,34]. The SCL-S-9 is a frequently applied screening instrument that assesses various MHP in adults. The nine items representing the dimensions somatization, obsessive-compulsive, interpersonal sensitivity, depression, anxiety, anger-hostility, phobic anxiety, paranoid ideation, and psychoticism were rated on a five-point scale ranging from 0 = *none at all* to 4 = *very severe*. We adapted the SCL-S-9 total score to the Global Severity Index of the SCL-90-R (GSI), which is the sum score over all 90 items of the SCL-90-R, and used the recommended gender-specific cutoff for the GSI (T-values ≥ 63) to identify parents with MHP [35].

Child MHP were identified by means of the child-reported Strengths and Difficulties Questionnaire (SDQ) [36], one of the most frequently used screening tools for psychological problems [37] in young people. The 20 items of the four problem scales (emotional problems, conduct problems, hyperactivity/inattention, peer relationship problems) were rated on a three-point-scale with 0 = *not true*, 1 = *somewhat true*, and 2 = *certainly true*. All items were summed up to generate the Total Difficulties score, with higher values indicating more severe problems. Following the normalized cut-off values by Meltzer and colleagues [23] we classified the Total Difficulties score into *normal*, *borderline*, and *abnormal* scores. The corresponding categorical variable was subsequently dichotomized with one group including only children and adolescents with *normal* scores and the second group including those with *borderline* and *abnormal* scores indicating MHP.

Sociodemographic factors, stressful life events, parental daily strain, family constellation, and parental chronic disease were assessed in addition to parental and child MHP. Parents reported on parental age, the socioeconomic status of the family (SES), unemployment, stressful life events, as well as on the family constellation, parental daily strain, and parental chronic disease.

Parental age was measured by asking the parent filling in the questionnaire to report on both the mother’s and the father’s age. The data were dichotomized into two groups consisting of mothers aged up to 44 years, and 45 years of age and older, and of fathers aged up to 49 years and 50 years of age and older, respectively.

The SES was measured using the Winkler-Index [38]. This score ranging from 3 to 21 points was computed using data on parental education, occupation, and household income. We used the metric variable for the multivariate analyses. For descriptive purposes and bivariate analyses, three groups were created based on the metric SES score differentiating those with low (3 to 8 points), medium (9–14 points), and high SES (15 to 21 points) [39].

Unemployment was assessed by an item enquiring about whether one or both of the parents are currently unemployed or not.

Four specific stressful live events were assessed by means of an index computed with four dichotomous items using both parent- and child-reported data. Children reported if a close person had died and if parents had separated or divorced within the last twelve months. The other two stressful live events included financial stress (reported by parents for children younger than 13 years and reported by children/adolescents themselves if aged 13 years or older) and an accident or severe illness happening to the child/adolescent (self-reported).

Parental daily strain was identified using an index made of 13 items enquiring about the burden caused by e.g., housekeeping, conflicts with the partner, parenting, tending a family member in need of care, job-related problems, or work-life balance in general. The response options for the items included in the index parental daily strain ranged from *not at all* to *very* strong. The created variable on parental daily strain was subsequently dichotomized with response options *strong* and *very strong* indicating high parental daily strain.

Difficult family constellation was measured using two dichotomous items assessing whether the parent was a single parent and whether the child or adolescent had three or more siblings.

Finally, the answer to whether either one or both parents suffer from a *chronic disease* was obtained through two items enquiring whether the parent filling in the questionnaire or the partner was suffering from a chronic disease or a disability (e.g., asthma, diabetes, rheumatism). The two items were subsequently integrated into one variable indicating chronic disease or disability in one or both parents.

### Statistical analysis

First analyses included frequencies, Odds Ratios, confidence intervals, χ^2^ tests and other tests of significance. The group of parents with MPH was compared to the group of parents without MHP with χ^2^ tests and Odds Ratio being calculated for all risk factors. In order to assess the association of all risk factors with parental MHP and the association of parental MHP with child MPH while simultaneously controlling for all other risk factors we conducted multiple logistic regression analyses.

Descriptive analyses were computed using weighted data. The weight was calculated based on data from the German microcensus with regard to the children’s age and gender, and the families’ SES. All statistical analyses were conducted using the software package IBM SPSS Version 22.0 [40].

## Results

### Sample

These present analyses rely on a subsample of *N* = 1158 families with children and adolescents aged 11 to 17 years, of which 47.2% (*n* = 547) were female. The mean age of the children and adolescents was 14.8 (*SD* = 1.76). The SES was distributed as follows: 16.0% (*n* = 185) of the families had a low, 65.5% (*n* = 758) a medium SES, and 18.5% (*n* = 215) of the families had a high SES. Only families with biological parents were included in our analyses. The prevalence of child MHP in the total sample was 9.8% (*n* = 113). In children of parents with MHP (*n* = 215), the mean age was 14.82 years, (*SD* = 1.89), and 47.9% (*n* = 103) were female. In children of parents without MHP (n = 943), the mean age was 14.77 (*SD* = 1.75), and 47.1% (*n* = 444) were female.

### Families with parental MHP compared with families without parental MHP

Parental MHP were identified in 18.6% (*n* = 215) of the total sample. The four most frequent MHP reported by the parents were depressive symptoms (22.6%, *n* = 262), interpersonal sensitivity (15.4%, *n* = 178), somatization (12.1%, *n* = 140), and obsessive-compulsive (11.2%, *n* = 130) (multiple answers possible).

Families with parental MHP are compared with families without MHP in Table 1. In families with parental MHP low SES was reported significantly more often and parents were significantly younger than in families without parental MHP. Parents with MHP also significantly more often were unemployed, showed higher parental daily strain, and reported two or more stressful life events. They were furthermore significantly more often single parents and significantly more often reported chronic diseases in one or both parents compared with parents without MHP. No difference between the two groups was found regarding the number of siblings in the family.

**Table 1:** Families with and without parental MHP and their children.

	Families with parental MHP *n* = 215 (18.6%)		Families without parental MHP *n* = 943 (81.4%)			
	*n*	(%)	*n*	(%)	*OR*	CI
**Child MHP**	41	(19.1)	72	(7.7)	2.85***	[1.88–4.32]
**SES**						
Low	60	(27.9)	125	(13.3)		
Medium	129	(60.0)	629	(66.6)	0.43***^a^	[0.30–0.61]
High	26	(12.1)	189	(20.1)	0.29***^b^	[0.17–0.48]
Age mother (< 45 yrs old)	145	(67.5)	505	(53.6)	0.56***	[0.41–0.76]
Age father (< 50 yrs old)	172	(80.2)	684	(72.5)	0.66*	[0.46–0.95]
**Risk Factors for MHP**						
Parental unemployment	31	(14.4)	57	(6.1)	2.62***	[1.64–4.17]
Two stressful life events or more	38	(17.9)	50	(5.3)	3.86***	[2.46–6.06]
Parental daily strain	106	(49.5)	226	(24.0)	3.11***	[2.29–4.23]
Single parent family	50	(23.1)	113	(12.0)	2.23***	[1.53–3.23]
Three siblings or more	15	(7.0)	41	(4.4)	1.64	[0.90–3.04]
Parental chronic disease	89	(41.3)	206	(21.9)	2.53***	[1.85–3.45]

***Note***. Weighted data; *N* = 1158

SES = Socioeconomic status

^a^ = medium SES versus low SES

^b^ = high SES versus low SES

*OR* = Odds Ratio; CI = Confidence Interval

*p ≤ .05.

**; *p* ≤ .01.

***; *p* < .001.

### Children of parents with MHP compared with children of parents without MHP

Significantly more children of parents with MHP reported MHP themselves (19.1%, *n* = 41) compared with 7.7% (*n* = 72) of children from parents without MHP: the risk for child MHP was about three times higher for children of parents with MHP compared to children of parents without MHP (*OR* = 2.85; CI [1.88 – 4.32]). Children of parents with MHP significantly more often reported having experienced two or more stressful life events compared with children of parents without MHP. More specifically, with respect to stressful life events, 15.8% (*n* = 34) of the children of parents with MHP experienced the death of a close person compared with 11.7% (*n* = 110); χ^2^(1) = 2.768, *p* = .096) of children of parents without MHP. Further, 12.8% (*n* = 28) of the children of parents with MHP experienced the divorce or separation of their parents compared with 4.2% (*n* = 40; χ^2^(1) = 24.426, *p* = .001) of children of parents without MHP. Moreover, 16.2% (*n* = 35) of the children of parents with MHP suffered of a severe illness or an accident and 18.2% (*n* = 39) reported financial difficulties compared with 10.5% (*n* = 99; χ^2^(1) = 5.718, *p* = .017) and 3.9% (*n* = 36; χ^2^(1) = 59.196, *p* = .001) of the children of parents without MHP. No differences between children of parents with or without MHP were found regarding child age and gender.

### Risk factors for parental MHP

The multivariate analysis (Table 2) revealed that of all investigated risk factors for parental MHP, the SES, parental unemployment, stressful life events, parental daily strain, parental chronic disease, and child MHP were significantly associated with parental MHP. Parental daily strain and parental chronic disease constituted the highest risks for parental MHP. The parents’ age, being a single parent family, and the existence of three or more siblings in the family were not significantly associated with parental MPH.

**Table 2:** Risk factors for parental MHP.

Risk Factors		*n*	Rates of Parental MHP ^a^ *n* (%)	*OR*	95% CI
Age mother	< 45 yrs old	650	145 (22.3)	0.86	[0.59–1.25]
	≥ 45 yrs old	508	70 (13.8)		
Age father	< 50 yrs old	856	172 (20.1)	1.01	[0.66–1.53]
	≥ 50 yrs old	302	43 (14.2)		
SES	Low	185	60 (32.4)	0.92**	[0.88–0.97]
	Medium	757	129 (17.0)		
	High	215	26 (12.1)		
Parental unemployment	No	1070	184 (17.2)	1.79*	[1.03–3.09]
	Yes	88	31 (35.2)		
Two stressful life events or more	< two SLE	1069	176 (16.5)	2.01*	[1.17–3.46]
	≥ two SLE	88	38 (43.2)		
Single parent family	No	995	165 (16.6)	1.35	[0.87–2.10]
	Yes	163	50 (30.7)		
Three siblings or more	< three siblings	1101	200 (18.2)	1.47	[0.78–2.76]
	≥ three siblings	56	15 (26.8)		
Parental daily strain	No	825	108 (13.1)	3.08***	[2.21–4.30]
	Yes	332	106 (31.9)		
Parental chronic disease	No	863	126 (14.6)	2.16***	[1.54–3.04]
	Yes	295	89 (30.2)		
Child mental health problems	No	1044	174 (16.7)	1.72*	[1.04–0.84]
	Yes	113	41 (36.3)		

***Note*.** *N* = 1164

^a^ = Weighted data; *OR* = Adjusted Odds Ratio; CI = Confidence Interval

SES = Socioeconomic status; SLE = Stressful life events

*p ≤ .05.

**p ≤ .01.

***p < .001, Model fit: χ^2^(10) = 117.67, *p* < .001; Nagelkerke *R*^*2*^ = 0.161.

### Risk factors for child mental health and the effect of parental MHP on child mental health

Table 3 shows the effect of parental MHP on the children’s mental health controlling for all other risk factors. The multivariate analysis revealed that female gender of the child as well as the mother’s older and the father’s younger age were significantly associated with child MHP. Moreover, having experienced two or more stressful life events and parental MHP were both significantly associated with child MHP. No associations were found between child age, SES, parental unemployment, parental daily strain, being a single parent family, having three siblings or more, and parental chronic disease.

**Table 3:** Risk factors for child MHP.

Risk Factors		*n*	Rates of Child MHP ^a^ *n* (%)	*OR*	95% CI
Gender child	Male	611	47 (7.7)	1.63*	[1.07–2.47]
	Female	547	67 (12.2)		
Age child	< mean age	489	61 (12.5)	0.92	[0.82–1.04]
	≥ mean age	668	52 (7.8)		
Age mother	< 45 yrs old	650	67 (10.3)	1.67*	[1.06–2.64]
	≥ 45 yrs old	507	46 (9.1)		
Age father	< 50 yrs old	856	92 (10.7)	0.48*	[0.27–0.84]
	≥ 50 yrs old	301	21 (7.0)		
SES	Low	186	26 (14.0)	0.95	[0.89–1.01]
	Medium	757	71 (9.4)		
	High	215	17 (7.9)		
Parental unemployment	No	1069	102 (9.5)	0.65	[0.28–1.52]
	Yes	88	11 (12.5)		
Two stressful life events or more	< two SLE	1069	91 (8.5)	2.38**	[1.27–4.46]
	≥ two SLE	89	23 (25.8)		
Single parent family	No	995	88 (8.8)	1.55	[0.88–2.71]
	Yes	162	25 (15.4)		
Three siblings or more	< three siblings	1101	107 (9.7)	1.46	[0.66–3.22]
	≥ three siblings	56	6 (10.7)		
Parental daily strain	No	826	89 (10.8)	0.81	[0.51–1.30]
	Yes	114	25 (7.5)		
Parental chronic disease	No	836	70 (8.1)	1.18	[0.75–1.87]
	Yes	294	43 (14.6)		
Parental mental health problems	No	942	72 (7.6)	1.74*	[1.06–2.86]
	Yes	215	41 (19.1)		

***Note*.** *N* = 1164

^a^ = Weighted data; *OR* = Adjusted Odds Ratio; CI = Confidence Interval

SES = Socioeconomic status; SLE = Stressful life events

*p ≤ .05.

**p ≤ .01; Model fit: χ^2^(12) = 37.087, *p* < .001; Nagelkerke *R*^*2*^ = .068.

## Discussion

This study presents population-based data on families with parental MHP and their children, stemming from the nationally representative BELLA study. About 19% of the parents in the total sample reported MHP. Children of parents with MHP reported MHP in about 19% compared with about 8% in children of parents without MHP (*OR* = 2.85). In multivariate analysis the risk for MHP in children of parents with MHP was nearly two times higher (*OR* = 1.72) compared to children of parents without MHP.

The prevalence for parental MHP in our study was lower than in former epidemiological studies. The 12-months-prevalence of adult mental disorders reported by Jacobi and colleagues [41] in Germany for example was almost 28%. This discrepancy could be attributed to the fact that we assessed MHP in parents during the past seven days only as provided by the SCL-S-9. The most frequent parental MHP in the present study were depressive symptoms (22.6%) and interpersonal sensitivity (15.4%), followed by somatization, and obsessive-compulsive symptoms (about 12% and 11%, respectively). These results are only to some extent in line with the most frequent adult mental health disorders by Jacobi and colleagues [41], who reported anxiety disorders in about 16% of the sample, followed by mood disorders in almost 10% of the cases, and obsessive-compulsive symptoms and somatization in 3.6% and 3.5% of the cases, respectively. By comparison, symptoms of anxiety among parents in our study were observed in 10.3% (*n* = 120) of the cases. But again, the instruments used to assess MHP differ considerably across both studies.

The prevalence of self-reported child MHP in the total sample was 9.8%. Again, this prevalence of MHP in children and adolescents is smaller than in other epidemiological studies, e.g. as reported by Barkmann and Schulte-Markwort [42] in their meta-analysis where the prevalence based on 33 German epidemiological studies was 17.6% for MHP in children and adolescents. Consistent with our results, the rates of child MHP at the BELLA study’s baseline assessment in children aged 11 to 17 years was 12.2% [22]. Again, the varying results for prevalence of MHP in children and adolescents in Germany have to be attributed to different measures and their respective time frames.

A rate of about 20% of the children of parents with MHP reported MHP. This percentage is remarkably lower than in studies that investigated children of parents with MHP in clinical samples, where about 50% of the offspring had MHP themselves [43,44]. Other epidemiological studies also report higher rates of MHP in the offspring of parents with MHP [11]. However, in the present study, children´s MHP were assessed using the children´s self-report, leading to lower rates of MHP compared with parent-reported MHP in the children (which were also surveyed but not presented). This bias is caused by the choice of the perspective and is a well-established phenomenon in psychosocial research [45]. We nevertheless decided to use the children´s self-reports in order to exclude potential bias due to parental symptomatology: Previous studies have shown that parents with MHP report on their children´s MHP heterogeneously [46,47]. Future studies may wish to investigate in more detail the effect of different perspectives on the prevalence of child MHP especially in children of parents with MHP. In line with previous epidemiological studies [11] we furthermore found that children of parents with MHP significantly more often reported MHP (19.1%) compared with children of parents without MHP (7.7%). This result is in line with the findings of Mc Laughlin and colleagues [48].

In our multivariate analysis on risk factors for parental MHP low SES, parental unemployment, stressful life events, parental daily strain, parental chronic disease, and child MHP were associated with parental MHP. Our results, stemming from an epidemiological sample, indicate that especially in families with parental MHP risk factors for MHP cluster together [10]. Therefore, these families represent a particular risk group for developing MHP. Moreover, our findings confirm the body of literature on risk factors for adult MHP and as established by previous studies [2,12–14,18,19].

Controlling for potential risk factors, our multivariate analysis on risk factors for child MHP demonstrated that the risk for children of parents with MHP to show MHP was still almost two times higher (*OR* = 1.74) than for children of parents without MHP. The model moreover revealed that female gender of the child, the age of the mother and the father, and having experienced two or more stressful life events were significantly associated with child MHP. A previous population based study on 13 to 17 year old adolescents by McLaughlin and colleagues also demonstrated that stressful life events were associated with a substantial proportion of child MHP [49]. We did not expect parental age to be associated with child MHP as we did not include very young or very old parents. As to our knowledge no previous studies have found similar results, the association of parental age and child MHP should be investigated in future research. Overall, our results on data of a population-based sample largely replicate former studies using clinical samples, which established that children of parents with MHP are a risk group for developing MHP [4,11].

Yet, the proportion of variance explained by our model was rather small. A-posteriori models using metric variables for the outcome as well as for predictors (if available) did not yield a higher proportion of explained variance. We also need to point out that this study’s purpose was to analyze risk factors only and therefore did not include protective factors such as personal, familial, or social resources. Yet, especially in adolescence, factors found outside the family such as the peer group or factors associated with school increase in importance, as explained by the literature published by developmental psychology for instance. We already published data on the effects of protective factors on children of parents with MHP aged 13 to 17 years: These analyses revealed that resources found outside of the family, such as personal and social resources (e.g., self-efficacy, school climate) contributed to the resiliency of children of parents with MHP [50].

Our findings indicate that the investigated risk factors for MHP do not have the same effects on parental mental health compared with the effects they have on child mental health. The majority of the investigated risk factors in the multivariate analyses on parental MHP were significantly associated with parental mental health. On the other hand, our regression analysis on child MHP revealed that the risk factors associated with child mental health were child female gender, parental age, stressful life events, and parental MHP only. These results firstly confirm the premise that adverse factors are more frequent in families with a parent suffering from MHP. They however also suggest that parental mental health has a greater influence on their child’s mental health compared to other potential risk factors, with the exception of stressful life events. This underlines the relevance of the parent-child relationship with respect to the mental health of the child, as outlined by Mattejat and Remschmidt and demonstrated by Beardslee and colleagues [3,51] by means of a preventive intervention focusing on the increased understanding between the child and the parent. The transmission of parental psychopathology to child MHP can partly be explained by maladaptive parent-child interaction patterns as demonstrated by van der Pol and colleagues [52]. Genetic aspects may also partly explain these results [7].

### Strengths and limitations

This study has a number of limitations: First, as it refers to cross-sectional data, the results do not allow us to draw causal conclusions. For instance, child MHP may have contributed to parental MHP, and vice versa, by means of self-reinforcing processes [19]. Studies using longitudinal designs would be able to disentangle this reciprocal association. Second, we were able to investigate only a certain number of risk factors, with the operationalization of certain risk factors not always being ideal, due to the design of the BELLA study. Future research should include further relevant risk factors for child MHP and use standardized scales throughout to assess the risk factors. Third, we were able to gather data on parental MHP by one parent only. Future studies should include data on parental mental health stemming from both parents.

However this study has also several strengths: First, whereas previous studies on children of parents with MHP were solely based on clinical samples–which are not representative for the whole of the population [19,53]–we have for the first time in Germany provided information on children of parents with MHP using a community sample. Second, previous studies mostly used clinical diagnoses of the parents to assess their mental health, whereas in the present study the SCL-S-9, a widely used screening tool for adult MHP, was applied. This could be deemed a limitation as clinical diagnoses are often referred to as the gold standard. However, with regard to early intervention and prevention programs, the screening tools that were used in the present study contribute to making the results more generalizable and the presented effects even more compelling. Third, we assessed child MHP by means of self-report in order to avoid a potential bias caused by parental MHP on the evaluation of child MHP [47]. However, using multiple perspectives always contributes to a better understanding of the data [54]. Future research should therefore include parent and teacher perspectives on child MHP.

## Conclusion

In Germany, almost 20% of the 11- to 17-year old children live with a parent with MHP. The risk for these children to report MHP themselves is nearly 2 times higher compared with children of parents without MHP. Furthermore, other investigated risk factors for child MHP such as parental daily strain, single parent family, or parental chronic disease do not seem to affect child mental health as much as parental MHP, with the exception of stressful live events. Families with parental MHP therefore represent a particular risk group for child MHP. Currently, different prevention and intervention programs for families with parental MHP are being developed and implemented in clinical settings for patients either in adult psychiatry or child and adolescent psychiatry. These programs show good effectiveness in reducing psychopathology and risk factors. [51,55,56]. The epidemiological data of the present study provides evidence that not only in clinical settings but also in the general population families with parental MHP show an increased risk for MHP. Consequently, our results should provide a basis for public health policy to identify families with MHP in the general population, to adapt clinical programs of prevention and intervention to the needs of these families, and to offer intervention and prevention programs in public health settings to families with parental MHP.

## Supporting information

S1 File Dataset. (XLS)
